# Normalization of circulating microRNA expression data obtained by quantitative real-time RT-PCR

**DOI:** 10.1093/bib/bbv056

**Published:** 2015-08-03

**Authors:** Francesco Marabita, Paola de Candia, Anna Torri, Jesper Tegnér, Sergio Abrignani, Riccardo L. Rossi

**Keywords:** normalization, circulating miRNA, qPCR, reference genes, geNorm, Normfinder

## Abstract

The high-throughput analysis of microRNAs (miRNAs) circulating within the blood of healthy and diseased individuals is an active area of biomarker research. Whereas quantitative real-time reverse transcription polymerase chain reaction (qPCR)-based methods are widely used, it is yet unresolved how the data should be normalized. Here, we show that a combination of different algorithms results in the identification of candidate reference miRNAs that can be exploited as normalizers, in both discovery and validation phases. Using the methodology considered here, we identify normalizers that are able to reduce nonbiological variation in the data and we present several case studies, to illustrate the relevance in the context of physiological or pathological scenarios. In conclusion, the discovery of stable reference miRNAs from high-throughput studies allows appropriate normalization of focused qPCR assays.

## Background

Over the past years, accumulating evidence started to support the use of microRNAs (miRNAs) circulating in blood as noninvasive biomarkers of disease conditions [1, 2]. These molecules can be extracted from serum, plasma and other body fluids and profiled through microarray, quantitative real-time reverse transcription polymerase chain reaction (qPCR) or sequencing [3]. Broadly speaking, it appears that in healthy conditions, miRNAs that are highly represented in the circulation are not enriched in specific families, and hence not enriched in specific biological functions, but rather originating from those mostly expressed by blood cells, endothelial cells or large and highly vascularized organs, such as liver, where considerable contacts exist between the cells and the blood [4–6]. Moreover, circulating miRNAs are differently enriched in different compartments: associated with exosomes (size 20–100 nm); microvesicles (size 0.2–1 µm); Argonaute proteins in a vesicle-free form; or high-density lipoproteins [7–9]. Notwithstanding their heterogeneous nature and origin, specific signatures of circulating miRNA have been associated to a variety of pathological conditions such as cancer [10–12], cardiovascular diseases [13, 14], diabetes [15], liver pathologies [16, 17] and sepsis [18, 19], among others (reviewed in [9, 20, 21]). Since the appearance of the first studies, it has been recognized that circulating miRNA levels do not get dramatically influenced by multiple freeze-thaw cycles, long-term storage or treatment with RNase before extraction [1, 2]. On the other hand, miRNA detection and quantification in either serum or plasma is heavily affected by pre-analytical and analytical challenges [22] that must be considered for miRNAs to be consistently suitable as clinical biomarkers.

A limitation of some of the reported works is the lack of a solid and common normalization strategy to account for inter-individual or intergroup variability. Most of the results were obtained by qPCR, using different reference genes for normalization. Typically, the use of ‘housekeeping’ genes for the normalization of qPCR data is straightforward because they represent endogenous controls that are affected by the same sources of variability as the target genes, during all the steps of the experimental pipeline. When quantifying *cellular* miRNAs, stable small RNA controls are currently used as reference RNAs. These include small noncoding RNAs, and specifically small nuclear RNA and small nucleolar RNA such as SNORD44 (RNU44), SNORD48 (RNU48) and RNU6-1 (Mamm U6). For *serum* miRNAs, there is growing evidence that the above-mentioned small RNAs are highly variable or not stably detectable [20, 23], thus leading to the search for suitable stable control miRNAs that are firmly detectable in human serum. The most common choice could be the use of a miRNA that does not vary considerably between individuals. However, this selection may be arbitrary when based on the variability of raw data only, without the assessment of the actual stability by more detailed ‘wet’ and ‘in silico’ analyses, especially for limited sample sizes. Picking a reference gene simply on the basis of no statistically significant difference between groups is considered insufficient to claim that the gene can be confidently considered as a stable reference. Indeed, for a given miRNA, the failure to reject the null hypothesis that there is no differential expression between the groups does not mean that the levels are the same between groups, according to the classical statistical hypothesis testing [24]. Therefore, a more complete evaluation is advisable, using *ad hoc* methods. While the identification of reference genes for qPCR normalization is a well-established process, the application of such methods to circulating miRNA data requires some methodological, experimental and analytical considerations, to reflect the different physiological nature of circulating miRNAs.

Here, we make some methodological considerations, then we review the terminology and explain the methods, and finally we present a few case studies, featuring the identification of reference miRNAs in several data sets, including sera from healthy and diseased individuals. Throughout this article, however, we do not advocate the adoption of a unique and common reference miRNA panel, which would be used universally across studies, but instead we recommend the use of a standardized methodology using data-driven approaches, for the establishment of candidate endogenous controls, which can be exploited as reference miRNAs.

## Methodological considerations

Plasma and serum are characterized by the presence of RNases and a low yield of RNA, which indeed cannot be measured by spectrophotometric determination. While microarray- and sequence-based approaches are currently available [25], qPCR has been the preferred one for the quantification of circulating miRNAs, given its sensitivity and specificity and high dynamic range [26]. High-throughput qPCR platforms exist for assaying multiple miRNAs in parallel, either with stem-loop reverse transcription (RT) reaction combined with TaqMan qPCR, or with a poly(A)-tailed RT combined with SYBR Green detection and LNA primers [26, 27]. The major limitation of this approach is that it cannot identify novel miRNAs, but for human studies, this factor can be less problematic, given the fact that human miRNA repertoire is well defined.

In the bloodstream, miRNAs have been shown to reside within a vesicle-associated fraction, including exosomes, microvescicles and apoptotic bodies [28] or protected by multi-protein complexes [7]. Although circulating miRNAs are consistently reported to be stable in serum after long-term storage and treatment with RNases or freeze/thaw cycles [1, 2, 6], they are influenced by compartmentalization, as vesicle-associated miRNAs seem to be more stable than those present in a non-membrane-bound form [29, 30]. Therefore, the measured variability is a result of true biological changes, which are associated with the phenomenon or disease of interest, and of technical, nonspecific variability, which is introduced during the multiple experimental steps. Potential sources of technical variability include: (a) the starting material and collection/isolation procedures (serum, plasma, exosomes, other vesicles); (b) multiple freeze-thaw cycles; (c) the RNA extraction method and its efficiency; (d) the input RNA quantity and quality; (e) the efficiency of the enzymatic reactions; and (f) the inter-individual variation in total RNA and miRNA concentration in plasma or serum. Therefore, the accuracy of the reported associations is dependent on a careful study design and appropriate data normalization. To ensure that proper measures are taken to minimize confounding factors, below we go through some considerations to guide the study design for the identification of reference circulating miRNAs.

- *Keep homogeneous conditions*. When using archived samples, select samples that have been processed according to the same standardized protocol, to reduce technical variation from this initial step in the data. For example, make sure to avoid hemolyzed serum specimens, as cellular RNA will contaminate the serum fraction and prevent the accurate determination of circulating miRNA profiles [31, 32]. Although plasma and isolated vesicles from blood represent an alternative starting material, mixing different collection methods should be obviously avoided, because the availability of circulating miRNAs in different biological compartments and distinctive efficiency and specificity will result in different miRNA identification and quantification [33, 34]. To further reduce experimentally induced variability, RNA extraction method must be thoroughly tested and standardized, as the yield of Trizol-based or column-based methods can result in qualitative and quantitative differences [35, 36].
- *Reduce freeze/thaw cycles*. Given that the ‘packaging’ of circulating miRNAs might influence their stability, it is possible that repeated freeze/thaw cycles of serum samples would differentially affect their levels [30] and negatively influence the identification of both stable and differentially expressed miRNAs.
- *Reduce batch effects*. A batch may be defined as a subgroup of samples or experiments, exhibiting a systematic nonbiological difference that is not correlated with the biological variables under study (for example, different experiment days, laboratory conditions, reagent lots or operators). Batch effects represent a common source of variability in high-throughput methods and should be avoided or reduced. Although methods exist [37–39] to reduce such unwanted variation, those methods fail when a biological variable is totally confounded with a batch variable. Nevertheless, when the experimental goal is to find normalizers, it is amenable not to mix different experimental batches.
- *Add a synthetic RNA for technical normalization*. The efficiency of RNA extraction, complementary DNA synthesis and PCR amplification can be monitored using an exogenous synthetic miRNA (for example, *Caenorhabditis elegans* cel-miR-39 or *Arabidopsis thaliana* ath-miR-159a), which are added as spike-in control during processing. When added to the lysis buffer during the extraction step, the synthetic RNA will be put through the same conditions as the endogenous miRNAs, hence providing a process control, for technical normalization [1]. However, this type of external reference will not be able to correct for other sources of variability, as for example the total concentration of the miRNA fraction in serum, which is likely changing inter-individually and/or in a disease-associated fashion. For a comprehensive normalization protocol, endogenous controls are required, either using global high-throughput-derived parameters (i.e. mean expression value per sample) or selected, validated stable reference miRNAs for focused assays. While miR-16 has been used to normalize the results in many studies, this miRNA was shown to be particularly susceptible to hemolysis, thus being far from an ideal choice as an internal control [22].

## Definitions

In fluorescence-based qPCR, the threshold cycle (*Ct*) is the PCR cycle number at which the fluorescence level meets the specified threshold (or *Cq*, according to the MIQE standard [40]). Relative Quantities (RQs) are here defined as those resulting after scaling *Ct* values with an external reference spike-in miRNA (i.e. ath-miR-159a or cel-miR-39-3p), and moving to the linear scale:

$$RQ=2^{-\Delta Ct}$$

$$\Delta Ct=Ct_{miRNA}-Ct_{spike}$$

The normalization factor (NF) is calculated as the geometric mean of the selected *k* normalizers, for each sample *j*. The NF is used to obtain the normalized relative quantities (NRQ) for each miRNA *i* and sample *j*:

$$NRQ_{ij}=\frac{RQ_{ij}}{NF_{j}}$$

Alternatively, NRQ may be obtained using a NF resulting from the geometric mean of RQs of all expressed miRNAs per sample, i.e. the mean obtained omitting detectors whose *Ct* is undetermined or above a specific threshold, that may vary according to the technical characteristics of the specific platform (i.e. *Ct* > 35).

## Selection of reference miRNAs

The effect of a normalization process is highlighting true biological changes and eliminating, or at least reducing, the variability introduced during the whole experimental process. In this section, we will overview available methods, we will give brief descriptions, and finally we will illustrate their application through case studies in the next section.

**Figure 1.**
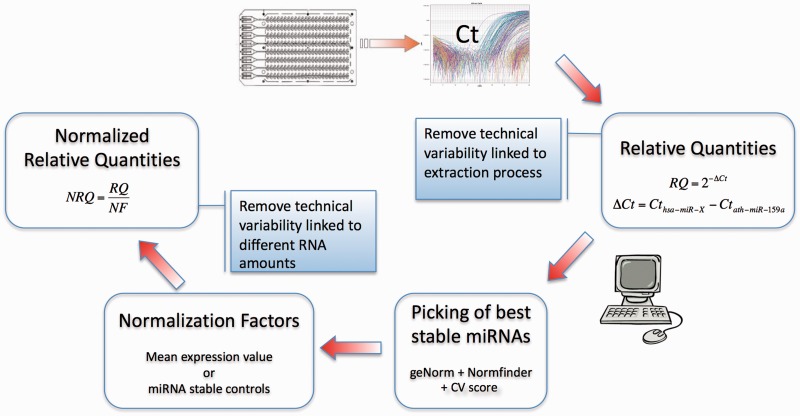
Schematic representation of the normalization workflow.

- *Exogenous controls*. The use of external synthetic reference miRNAs has been documented starting from the early works on circulating miRNAs [1], where they have been spiked at the time of RNA isolation to correct for the differences in yield between samples. This point has been discussed in the previous section.
- *Endogenous controls*. Other normalization approaches, including endogenous controls have been exploited. For array-based approaches, a normalization method based on the mean expression value of all miRNAs has been proposed and validated for qPCR data [41]. Other data-driven methods are practicable and they have been evaluated before [42], i.e. quantile normalization [43] or rank-invariant set normalization [44], for profiling experiments that include from hundred to thousand parallel measurements. Because these strategies are only applicable when high-throughput miRNA profiling is performed, obtaining a number of solid normalizers is extremely advantageous, considering the prerequisite of reducing the number of reference miRNAs and increasing the throughput and the feasibility of single or focused qPCR assays. Therefore, the search of candidate reference miRNAs, using a combination of validated bioinformatic approaches, represents a first step in the establishment of large scale, prospective or retrospective studies, which will evaluate a limited panel of circulating miRNAs, but in a wide cohort.
- *Data quality control*. When the goal is to identify stable miRNAs, major source of technical variability should be reduced to a minimum. For instance, outliers resulting from artificial and/or non-exponential amplification profile should be identified and removed, through visual inspection of the amplification curves or inspection of instrument-provided flags. Typically, extreme outlier *Ct* values arise from an abnormal amplification plot that crosses the threshold but does not reflect a real amplification, resulting in a particularly low *Ct*. This procedure is reasonably crucial in the analysis pipeline because certain quantities derived from the data may be strongly influenced by the presence of such extreme outliers, like the mean or the sum of the RQs.
- *Missing values*. High-throughput data may often contain missing values. A simple solution would be considering only miRNAs with complete observations. The rationale behind this hard filtering would be the following: circulating miRNAs serving as endogenous controls should be abundantly and stably detected in all samples to be considered for analysis. Therefore, the missed detection of a specific miRNA in a subgroup of samples would undermine its ‘candidature’ as normalizer. Alternatively, a missing value could be generated from technical factors other than biological reasons in an exiguous number of samples, but the corresponding miRNAs could be reasonably included in the analysis. Because some of the used functions could not handle missing values, they can be imputed using an appropriate method, like the k-nearest neighbor method in the impute R library (http://www.bioconductor.org/packages/release/bioc/html/impute.html).
- *Algorithms for the identification of stable miRNAs*. Different algorithms can be used to reveal the best internal reference miRNAs, including geNorm [45] and Normfinder [46]. Other approaches are available, including the BestKeeper algorithm [47] and the Szabo *et al*. method [48]. Here, we describe the use of a combination of the two most widely used methods, namely geNorm and Normfinder, and a simpler scoring algorithm based on the Coefficient of Variation (CV) score, outlined here, which represents a conceptually simple way to describe the stability, and it is similar to what has been previously used [41, 47, 49, 50]. The workflow is summarized in Figure 1.
   geNorm and Normdinder can be conveniently run in R with the SLqPCR library (http://www.bioconductor.org/packages/release/bioc/html/SLqPCR.html) or script (http://moma.dk/normfinder-software), respectively, or other Bioconductor packages (NormqPCR). geNorm is an algorithm to reveal the most stable reference genes from a list of candidates, which have been quantified in a group of samples. For each miRNA, the algorithm calculates a pairwise variation (V) with all other miRNAs across the samples, as the SD of the log-transformed expression ratios. Then, a stability score (M) is defined as the average V of a miRNA with all other miRNAs. The less stable miRNA (highest M) is removed, and the cycle is repeated until the most stable pair has been obtained. Normfinder is a model-based approach to estimate intergroup and intragroup variances of the log-transformed expression ratios, and then combine them into a stability value, which summarizes the variability and therefore represents a measure of the systematic error for the candidate miRNA.
   The CV score algorithm is based on the comparison of the variability of the miRNAs across samples, after accounting for the total miRNA recovery for each sample. It is described as follows: from the matrix of RQ (*m* detectors, *n* samples), calculate a new matrix *X* for miRNA *i* and sample *j* where:
  $$X_{ij}=\frac{RQ_{ij}}{\sum_{i=1}^{m}RQ_{ij}}$$
   Then, for each miRNA *i*, calculate the CV of X_i*_, as its SD divided by the mean. This parameter is useful to evaluate stability across samples. The sum of all RQ for each sample *j* can be considered a surrogate of the total miRNA amount, and therefore, scaling the RQs by the total miRNA quantities obtained from the profiling represents a precaution against the possibility of different input amounts, which would invalidate the use of the CV as a measure of stability. As a consequence, the lesser the CV of X_i*_, the better the miRNA *i* may be considered as normalizer. By definition, a normalizer must be correlated to the total miRNA amount.

(vi) Selection of candidate miRNAs. The algorithms above generate a score that represents the stability of the candidate miRNAs in the expression data set, usually in such a way that a smaller score corresponds to higher expression stability. The top candidate miRNAs should be selected among those performing well in all the methods. To summarize the results, the distance from the origin in *n*-dimensional space is proposed here as a metric, or summarized stability score (SSS). Assuming three methods have been used (geNorm, Normfinder, CV score), the SSS score would be:
  $$SSS=\sqrt{{\left(Score_{geNorm}\right)}^{2}+{\left(Score_{Normfinder}\right)}^{2}+{\left(Score_{CV}\right)}^{2}}$$
   Ranking SSS in ascending order, the final miRNA reference list is obtained. Finally, a gene expression NF is calculated for each sample based on the geometric mean of a user-defined number of reference genes, as proposed early [41, 45, 50].

(vii) *Validation*. The ideal validation of the reference circulating miRNAs would require an additional independent experimental profiling. However, when this option is either out of scope or not possible, at least an independent computational approach could be used to confirm the ranking. Principal component analysis (PCA) represents a valuable and independent approach for classifying the miRNAs on the basis of their expression profile. The analysis of unscaled data takes into account the extent of the changes in a relative expression data set, which is usually reported by the first principal component (PC). Therefore, the most informative components are usually PC2 and PC3. Whereas, for autoscaled data, a PC1 versus PC2 plot is helpful in identifying and evaluating reference miRNAs [51]. Autoscaled RQs for miRNA *i* and sample *j* are defined as follows:
   
  $$ARQ_{ij}=\frac{\log_{10}RQ_{ij}-mean(\log_{10}RQ_{i*})}{st.dev(\log_{10}RQ_{i*})},$$
  where *mean*(log_10_RQ_i*_) and *st.dev*(log_10_RQ_i*_) are the mean and the SD of log_10_(RQ), respectively. Autoscaled data have mean 0 and SD of 1.

## Reference miRNA evaluation: case studies

*Case study 1: Liver pathology.* As a case study analysis, we present here the identification of stable reference miRNAs in a collection of frozen sera from either healthy controls or patients with Hepatitis C infection, including samples at different disease stages—chronic hepatitis, liver cirrhosis or hepatocellular carcinoma (Marabita *et al*., in preparation). The ultimate goal of data normalization is ensuring an adequate reduction of the experimentally induced variability. For this purpose, a two-level normalization strategy was used. Firstly, we used a synthetic miRNA (ath-miR-159a) as spike-in control, within the RNA lysis buffer, at a suitable concentration (5 pM). With the expression of the miRNA levels relative to this external reference, we provided a quality control for differences in extraction or RT efficiencies. In our laboratory experience, the yield of RNA from small volume plasma or serum samples (i.e. 100–200 μl) is below the limit of accurate quantification by spectrophotometry, a finding that is confirmed by other groups [52]. Therefore, a normalization based on a fixed amount of starting RNA is routinely impracticable. The use of a fixed volume of starting RNA is amenable, although the relationship between the total RNA amount in serum and the corresponding miRNA fraction has not been described. To further reduce experimental variability, we also used an endogenous control to eliminate variability linked to different quality and quantity of the starting RNA amount. This endogenous reference was either a global parameter, such as the mean of all expressed miRNAs, or a NF calculated from the levels of the reference miRNAs.

**Figure 2.**
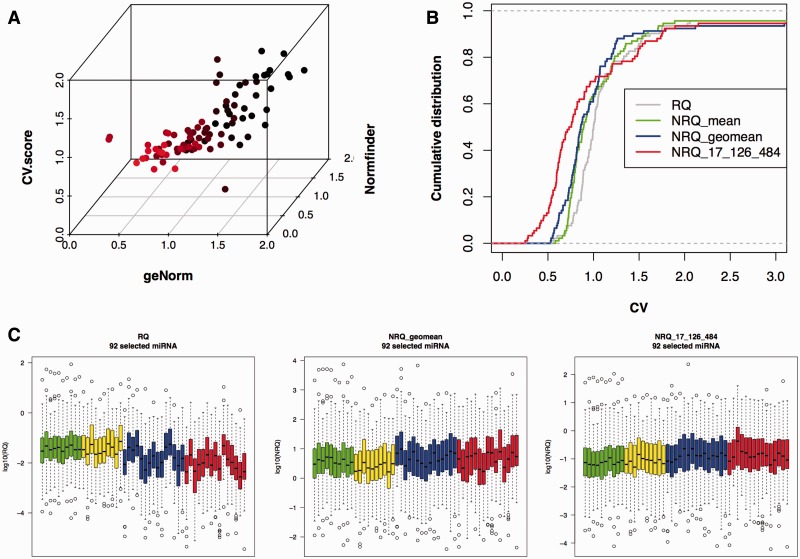
Case study 1: Identification of stable normalizers and reduction of variability. (A) The three scores presented here are shown in a 3D scatterplot. (B) A cumulative distribution plot shows the reduction of the technical variability. The CV of RQ or NRQ was calculated for miRNAs detected in at least two-thirds of the samples. Data presented in the plot are either not normalized (RQ, gray line), normalized with the arithmetic mean (NRQ_mean, yellow line), geometric mean (NRQ_geomean, blue line) or with three stable controls (NRQ_17_126_484, red line). The left-shifted curves show a reduction on variability. (C) Box plots showing the distribution of RQ or NRQ for each sample, before and after normalization, for miRNAs detected in at least two-thirds of the samples. Each sample is colored according its biological group (green: healthy controls, yellow: chronic hepatitis, blue: liver cirrhosis, red: hepatocellular carcinoma). A colour version of this figure is available at BIB online: http://bib.oxfordjournals.org.

**Figure 3.**
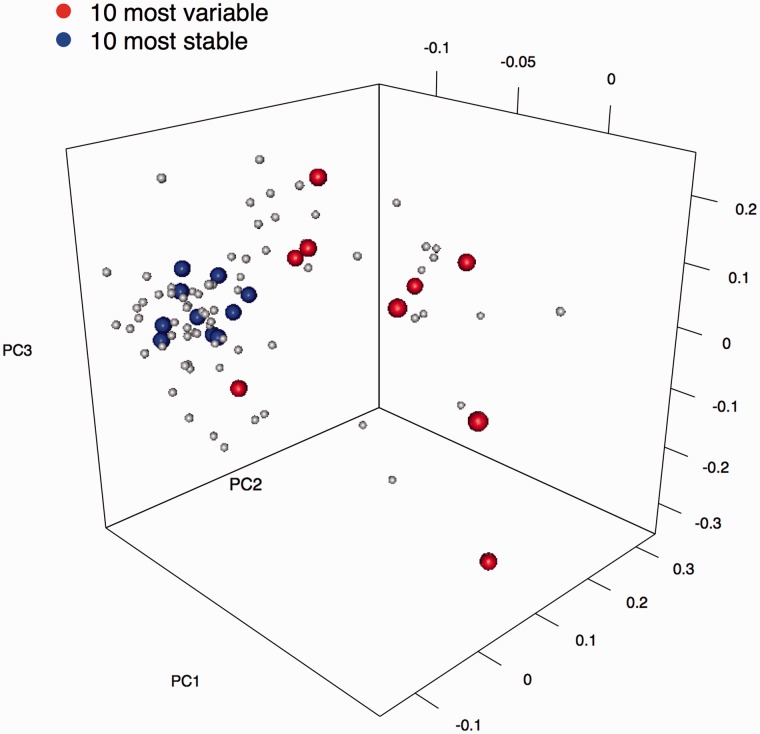
Case study 1: Validation of reference miRNA selection with PCA. PCA with autoscaled data shows independently that the combination of the presented algorithms is able to successfully identify stable miRNAs, which are grouped according to their variability. Blue spheres correspond to the top 10 stable miRNAs, while the red spheres correspond to the 10 most variable, according to the SSS. A colour version of this figure is available at BIB online: http://bib.oxfordjournals.org.

The three different algorithms described above (geNorm, Normfinder, CV score) were used to reveal the best internal reference miRNAs. An RQ matrix containing the values of miRNAs detected in at least two-third of samples per group, plus the arithmetic mean, the geometric mean and the median value of RQs was used as input. The final ranking was obtained summarizing the three scores, i.e. calculating the SSS in a three-dimensional space (Figure 2A and Supplementary Table S1). There was a good agreement between the three methods (multiple R^2 ^= 0.71), suggesting that an accurate selection of low-scored miRNAs can be used to calculate the NF. To pick the reference miRNAs, we assigned them to the respective families using miRClassify [53], with the goal of including only one element from the same miRNA family as accomplished for intracellular miRNAs [50] because members of the same miRNA family share sequence similarities and hence possible functional overlap [54]. NRQs were obtained using the geometric mean of three miRNAs (hsa-miR-17, hsa-miR-126, hsa-miR-484) or, alternatively, NRQs were obtained using the global arithmetic or geometric mean of all expressed miRNAs per sample. miRNAs showing a normalization performance similar to the mean expression value have been proposed as candidate normalizers [41]. Hence, a combination of reference miRNAs may be suitable in reducing data variation. To test this hypothesis, the effective reduction in data variability was evaluated looking at the cumulative distribution of the CV of NRQ and not normalized data (Figure 2B). Finally, the two alternative normalization strategies based on either the global geometric mean or the NF resulting from three top miRNAs were compared. The distribution of miRNA expression before and after normalization is shown in Figure 2C. Furthermore, candidate normalizers were tested and evaluated by PCA. Figure 3 shows the PCA with autoscaled data. Interestingly, miRNAs scoring as the most stable ones were clustered together at the narrow end of a funnel-like structure, showing that they are less variable with respect to the others (blue dots in Figure 3).

The procedure presented above can be generalized and in principle may reveal the candidate stable endogenous controls in a variety of experimental setting involving circulating miRNAs, provided that an external reference miRNA has been spiked in and then measured. We will illustrate below further examples of the application of the procedure in two other biological contexts.

*Case study 2: Vaccinated healthy donors.* A corresponding analysis was performed on a data set comprising 12 pooled sera samples, each from five individuals, and contrasting circulating miRNAs from healthy donors, at baseline and after influenza vaccination [34]. We applied the above-described three methods, confirming a good concordance (multiple R^2 ^= 0.89, Supplementary Figure S1A), and ranked the miRNAs according to their decreasing SSS (Supplementary Table S2). Here, we selected hsa-miR-146 b-5p, hsa-miR-142-3p and hsa-miR-24 as reference. Once more, we obtained a reduction in data variability as shown by the cumulative distribution of the coefficients of variation (Supplementary Figure S1B). The corresponding PCA plots also confirmed the grouping of stable versus variable miRNAs.

*Case study 3: Crohn’s disease.* Serum samples from patients with Crohn’s disease, either on remission or during the active phase, were assayed for circulating miRNAs, and the evaluation of endogenous controls was performed as above, ranking the miRNAs by decreasing stability (Supplementary Table S3). The results are shown in Supplementary Figure S2A–C. Using *Ct* values from an independent sample set, including sera form patients with Crohn’s disease [55], we confirmed that the top stable miRNAs had a corresponding smaller variation, as compared with the bottom variable miRNAs, hence confirming that the method is instrumental in reliably selecting endogenous controls for qPCR normalization.

**Figure 4.**
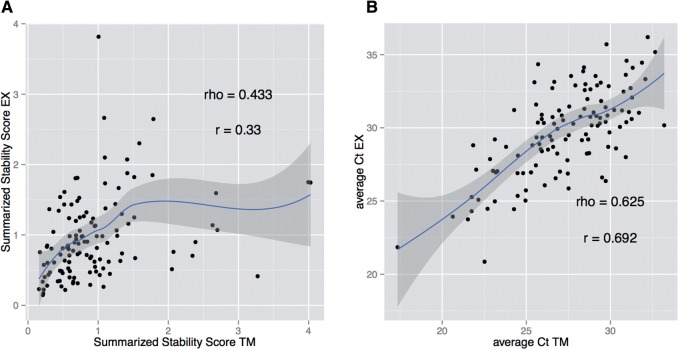
Case study 4: Platform comparison. (A) The SSS obtained with the two platforms is shown. Only miRNAs assayable and detected with both platforms in all samples were included. A loess smoothing and its confidence interval are shown. (B) Average *Ct* values are shown together with a loess smoothing and its confidence intervals.

*Case study 4: Healthy donors, different profiling platforms.* Several platforms are available for quantification of circulating miRNAs, and variation in performance exists between them. Even for qPCR-based assays, difference exists in term of accuracy, reproducibility, sensitivity and specificity [3]. Therefore, we extended the analysis to investigate whether the estimation of miRNA stability could be influenced by the choice of the commercial platform. We applied our algorithm to a data set obtained, profiling the same RNA from five healthy donors with two commercial platforms (TM: Life Technology TaqMan miRNA cards and EX: Exiqon miRCURY LNA) [34]. Given the different miRNA content, we selected miRNAs assayable and detected with both platforms and in all samples. Moreover, we adapted the above algorithm to calculate RQs with respect to the highest expressed miRNA (minimum *Ct* value), because the same external reference miRNA cannot be quantified with the two methods. When running two independent computations to calculate the miRNA stability score, we observed partial correlation between the platforms (Pearson *r* = 0.33, Spearman *rho* = 0.43, Figure 4A), despite the corresponding average *Ct* values being better correlated (Pearson *r* = 0.69, Spearman *rho* = 0.63, Figure 4B). However, the pipeline performed well as before (Supplementary Figure S3A), stable miRNAs were straightforwardly separated in the PCA space (Supplementary Figure S3B and C) and a general agreement with the relative ranking was observed, as top-scoring miRNAs had lower ranks with both platforms (Supplementary Table S4), with notable exceptions. For instance, hsa-miR-223 was scored as the most stable with TM, but performed poorly with EX. Possible explanations include different specificities and cross-reactivity for the two platforms. We finally calculated a combined score to select normalizers and, using the top three stable miRNAs (hsa-miR-484, hsa-miR-24 and hsa-miR-126), we verified that we obtained the usual reduction in data variability as shown by the cumulative distribution of the coefficients of variation (Supplementary Figure S3D and E), although to a different extent for the two groups.

## Conclusion

In the analysis of circulating miRNAs, various strategies have been used for data normalization, ranging from the use of miRNA or small RNA controls, external spike-in molecules or measures of central tendency. These substantial differences can possibly bias the ability of identifying differences between groups and certainly represent a major disadvantage in comparing expression levels between different studies. In addition to the standardization of sample collection and processing, the use of an accurate normalization procedure will ensure better reproducibility, and the identification of endogenous reference miRNAs will empower the feasibility of large-scale assay on selected markers.

A survey of best-scored reference miRNAs is shown in Supplementary Figure S4, along with the other three measurements (median, mean and geometric mean). Central tendency measures resulted in optimal internal references, as previously reported [41]. The normalization performance, measured as stability score (the lower the better), is not constant under different conditions (case studies) and for diverse NFs. Thus, although it is tempting to propose ‘universal’ reference miRNAs, care should be used when interpreting the results. In our view, the tendency to select a priori universal normalizers for circulating miRNAs should be substituted in favor of the adoption of standardized methods to select case-specific normalizers, because biases or inaccuracies in the relative quantification of circulating miRNAs can result from the choice of the reference gene(s) [56]. Indeed, there is a need for the development and adoption of an effective approach in serum miRNA normalization. We have suggested guidelines, including experimental and bioinformatics aspects, with the goal of guiding the experimental design and the analytical steps in the identification of reference miRNAs.

A number of reasons hold against the adoption of a universal reference circulating miRNAs. Firstly, the compartmentalization of circulating miRNAs, although facilitating the purification of specific isolates, hinders the clear identification of a universal ‘housekeeping’ miRNA that would be detected at a constant level across studies and conditions, as different preparation methods may result in different relative enrichment for the different compartments (membranous versus protein-bounded) [29, 30]. Secondly, miRNA expression has been shown to be variable for different tissues, development stages and disease conditions [57, 58], and therefore a possible covariate is the inclusion in the analysis of only sera from healthy controls or a combination of patients and healthy controls, because the profile of circulating miRNAs in a specific pathological condition could be different from the corresponding normal controls. Finally, the precise biological phenomena by which miRNAs are released into the bloodstream is not entirely elucidated [21], although is it is reasonable to assume that sources of variability may include both expression changes within the originating tissue and differential release within the blood circulation. The latter mechanism is accounted for a combination of passive release, mainly related to cell death, and controlled release, mainly through exosomes and microvesicles containing miRNAs.

For all the above-mentioned reasons, although the definition of a normalizer miRNA with broad applicability would be advantageous, we suggest switching the focus from the normalizers to the normalization method. From a practical point of view, potential normalizers should always be evaluated in a pilot phase, using a conserved methodology—such as the one we propose— to verify that in the each specific contrast of interest, they show optimal stability. With the procedure proposed in this article, we aim at identifying the best study-specific normalizers and therefore flattening the unavoidable bias introduced by the operational methods in each laboratory. The provision of uniform procedural guidelines—more than a universal set of normalizers—will assist in obtaining reliable quantifications and comparisons of circulating miRNA. These guidelines come at the cost of rejecting generalization in favor of reliability.

Key Points

- Studies on circulating miRNAs represent an active research field, and examples exist in the literature showing their potential use as diagnostic biomarkers. Most of the findings have been obtained by quantitative real-time RT-PCR; however, there is no consensus on the normalization process.
- The nature of the data obtained by assaying circulating miRNAs poses new challenges, including the unavailability of validated universal reference miRNAs, which are stably detected in the bloodstream and display broad applicability. Because of the lack of a common normalization framework, the results obtained by different laboratories may be incomparable directly or even contradictory, thus hindering systematic review or meta-analysis.
- In our article, we propose switching the focus from the normalizers to the normalization method: the tendency to select a priori universal normalizers for circulating miRNAs should be substituted in favor of the adoption of standardized methods to select case-specific normalizers. We suggest guidelines, including experimental and bioinformatics aspects, and present case studies that describe the applicability of the reviewed approaches, with the goal of guiding the experimental design and the analytical steps in the identification of reference miRNAs.

## Supplementary Data

Supplementary data are available online at http://bib.oxfordjournals.org/.

Supplementary Data
