# Supplementary material for: Normalization of circulating microRNA expression data obtained by quantitative real-time RT-PCR
Source: Brief Bioinform. 2015 Aug 3;17(2):204–12. doi: 10.1093/bib/bbv056 (PMC4793896; doi:10.1093/bib/bbv056)
Supplement: Supplementary Data [file supp_17_2_204__index.html]

Normalization of circulating microRNA expression data obtained by quantitative real-time RT-PCR — Normalization of circulating microRNA expression data obtained by quantitative real-time RT-PCR — Supplementary Data 

# Normalization of circulating microRNA expression data obtained by quantitative real-time RT-PCR

## Supplementary Data

files

- Supplementary Data - zip file
