# Supplementary material for: Normalization of circulating microRNA expression data obtained by quantitative real-time RT-PCR
Source: Brief Bioinform. 2015 Aug 3;17(2):204–12. doi: 10.1093/bib/bbv056 (PMC4793896; doi:10.1093/bib/bbv056)
Supplement: Supplementary Data [file supp_bbv056_suppl_data.zip › supp_tables.docx]

**Supplementary table 1.** Stable serum miRNAs in healthy controls and HCV-associated liver pathologies. Only the top ten miRNA are shown.

|  | ***geNorm*** | ***Normfinder*** | ***CV score*** | ***Mean Ct*** | ***SSS*** |
| --- | --- | --- | --- | --- | --- |
| ***hsa-miR-126*** | 0.579 | 0.32 | 0.773 | 25.0 | 1.017 |
| ***hsa-miR-17*** | 0.735 | 0.39 | 0.656 | 25.0 | 1.059 |
| ***hsa-miR-30c*** | 0.633 | 0.35 | 0.813 | 26.8 | 1.088 |
| ***hsa-miR-30b*** | 0.649 | 0.4 | 0.797 | 26.8 | 1.103 |
| ***median*** | 0.254 | 0.46 | 1.008 | NA | 1.137 |
| ***hsa-miR-20a*** | 0.781 | 0.53 | 0.679 | 25.6 | 1.162 |
| ***geomean*** | 0.254 | 0.49 | 1.025 | NA | 1.164 |
| ***hsa-miR-484*** | 0.704 | 0.57 | 0.739 | 25.8 | 1.169 |
| ***hsa-miR-342-3p*** | 0.681 | 0.37 | 0.903 | 27.8 | 1.19 |
| ***hsa-miR-146a*** | 0.767 | 0.54 | 0.770 | 25.8 | 1.214 |
| ***hsa-miR-106a*** | 0.956 | 0.36 | 0.677 | 25.1 | 1.226 |
| ***hsa-miR-320*** | 0.666 | 0.41 | 0.961 | 26.1 | 1.238 |

**Supplementary table 2.** Stable serum miRNAs in vaccinated healthy donors. Only the top ten miRNA are shown.

|  | ***geNorm*** | ***Normfinder*** | ***CV score*** | ***mean Ct*** | ***SSS*** |
| --- | --- | --- | --- | --- | --- |
| ***Mean*** | 0.254 | 0.09 | 0.071 | NA | 0.279 |
| ***hsa-miR-146b-5p*** | 0.173 | 0.1 | 0.203 | 26.8 | 0.285 |
| ***hsa-miR-142-3p*** | 0.184 | 0.06 | 0.212 | 24.8 | 0.287 |
| ***hsa-miR-24*** | 0.191 | 0.09 | 0.195 | 22.3 | 0.287 |
| ***hsa-miR-191*** | 0.268 | 0.12 | 0.109 | 22.5 | 0.313 |
| ***hsa-miR-106a*** | 0.173 | 0.1 | 0.247 | 22.2 | 0.318 |
| ***hsa-miR-19a*** | 0.250 | 0.13 | 0.160 | 25.93 | 0.323 |
| ***hsa-miR-19b*** | 0.241 | 0.12 | 0.189 | 21.0 | 0.329 |
| ***hsa-miR-26a*** | 0.263 | 0.11 | 0.169 | 24.7 | 0.331 |
| ***hsa-miR-92a*** | 0.220 | 0.11 | 0.234 | 21.4 | 0.339 |
| ***hsa-miR-484*** | 0.274 | 0.15 | 0.138 | 22.1 | 0.342 |

**Supplementary table 3.** Stable serum miRNAs in Crohn’s disease. Only the top ten miRNA are shown.

|  | ***geNorm*** | ***Normfinder*** | ***CV score*** | ***Mean Ct*** | ***Summary score*** |
| --- | --- | --- | --- | --- | --- |
| ***geomean*** | 0.157 | 0.13 | 0.432 | 0.478 | NA |
| ***mean*** | 0.448 | 0.08 | 0.145 | 0.478 | NA |
| ***median*** | 0.157 | 0.15 | 0.465 | 0.513 | NA |
| ***hsa-miR-135a**** | 0.331 | 0.13 | 0.394 | 0.531 | 23.0 |
| ***hsa-miR-106a*** | 0.5 | 0.15 | 0.37 | 0.64 | 24.0 |
| ***hsa-miR-17*** | 0.544 | 0.2 | 0.467 | 0.744 | 24.0 |
| ***hsa-miR-21*** | 0.649 | 0.17 | 0.391 | 0.777 | 27.0 |
| ***hsa-miR-30a*** | 0.621 | 0.2 | 0.467 | 0.802 | 26.9 |
| ***hsa-miR-30e**** | 0.582 | 0.18 | 0.548 | 0.819 | 30.9 |
| ***hsa-miR-19b*** | 0.676 | 0.21 | 0.413 | 0.82 | 23.0 |
| ***hsa-miR-222*** | 0.767 | 0.18 | 0.355 | 0.864 | 27.3 |
| ***hsa-miR-106b*** | 0.701 | 0.24 | 0.502 | 0.894 | 27.4 |
| ***hsa-miR-484*** | 0.738 | 0.24 | 0.459 | 0.901 | 24.5 |

**Supplementary table 4.** Stable serum miRNAs in healthy donors, using different qPCR platforms (TaqMan vs Exiqon). Only the top ten miRNA are shown.

|  | ***TM*** | | | | | ***EX*** | | | | |  |
| --- | --- | --- | --- | --- | --- | --- | --- | --- | --- | --- | --- |
|  | ***geNorm*** | ***Normfinder*** | ***CV score*** | ***Mean Ct*** | ***SSS*** | ***geNorm*** | ***Normfinder*** | ***CV score*** | ***Mean Ct*** | ***SSS*** | ***SSS6**** |
| ***geomean*** | 0.168 | 0.05 | 0.117 | NA | 0.211 | 0.13 | 0.06 | 0.04 | NA | 0.149 | 0.258 |
| ***hsa-miR-484*** | 0.11 | 0.16 | 0.095 | 22.8 | 0.217 | 0.117 | 0.09 | 0.082 | 27.872 | 0.169 | 0.275 |
| ***mean*** | 0.069 | 0.14 | 0.01 | NA | 0.157 | 0.212 | 0.09 | 0.044 | NA | 0.234 | 0.282 |
| ***median*** | 0.174 | 0.06 | 0.126 | NA | 0.223 | 0.146 | 0.12 | 0.124 | NA | 0.226 | 0.318 |
| ***hsa-miR-24*** | 0.179 | 0.13 | 0.159 | 22.2 | 0.272 | 0.229 | 0.13 | 0.102 | 25.08 | 0.282 | 0.392 |
| ***hsa-miR-126*** | 0.069 | 0.19 | 0.055 | 21.7 | 0.21 | 0.17 | 0.22 | 0.192 | 24.318 | 0.338 | 0.398 |
| ***hsa-miR-320*** | 0.135 | 0.13 | 0.145 | 24.5 | 0.237 | 0.259 | 0.25 | 0.189 | 26.923 | 0.407 | 0.471 |
| ***hsa-miR-26a*** | 0.162 | 0.19 | 0.152 | 24.5 | 0.293 | 0.246 | 0.28 | 0.235 | 25.361 | 0.44 | 0.529 |
| ***hsa-miR-766*** | 0.228 | 0.33 | 0.142 | 26.9 | 0.425 | 0.202 | 0.21 | 0.181 | 29.933 | 0.343 | 0.547 |
| ***hsa-miR-101*** | 0.239 | 0.37 | 0.182 | 29.7 | 0.477 | 0.217 | 0.13 | 0.09 | 26.375 | 0.269 | 0.547 |
| ***hsa-miR-151-3p*** | 0.208 | 0.21 | 0.105 | 25.8 | 0.314 | 0.279 | 0.31 | 0.224 | 28.894 | 0.474 | 0.568 |
| ***hsa-miR-93*** | 0.234 | 0.35 | 0.171 | 25.0 | 0.454 | 0.181 | 0.23 | 0.189 | 25.735 | 0.348 | 0.572 |
| ***hsa-miR-106a*** | 0.274 | 0.41 | 0.196 | 21.8 | 0.531 | 0.117 | 0.14 | 0.124 | 25.286 | 0.221 | 0.575 |

* Obtained combining the six individual scores
