# Supplementary material for: Normalization of circulating microRNA expression data obtained by quantitative real-time RT-PCR
Source: Brief Bioinform. 2015 Aug 3;17(2):204–12. doi: 10.1093/bib/bbv056 (PMC4793896; doi:10.1093/bib/bbv056)
Supplement: Supplementary Data [file supp_bbv056_suppl_data.zip › Supp_Figures_miRNA_norm.pdf]

Figure S1. Case study 2: Vaccinated healthy donors. A) The three scores presented in the text are shown in a 3D scatterplot. **B)** Cumulative distribution plot of the CVs of the analyzed miRNAs. The presented data are either not normalized (RQ, grey line), normalized with the arithmetic mean (NRQ_mean, yellow line), geometric mean (NRQ_geomean, blue line) or with three stable controls (NRQ_ref, red line). The left-shifted curves shows a reduction on variability. **C)** PCA as an independent way of grouping miRNAs based on their variability. The top ten stable miRNA are shown as blue spheres, while the red sphere correspond to the ten most variable, according to the SSS.

Figure S2. Case study 3: Crohn's disease. A) The three scores presented in the text are shown in a 3D scatterplot. **B)** Cumulative distribution plot of the CVs of the analyzed miRNAs. The presented data are either not normalized (RQ, grey line), normalized with the arithmetic mean (NRQ_mean, yellow line), geometric mean (NRQ_geomean, blue line) or with three stable controls (NRQ_ref, red line). The magnitude of the left-shifing between curves shows a minor reduction on variability. **C)** PCA as an independent way of grouping miRNAs based on their variability. The top ten stable miRNA are shown as blue spheres, while the red sphere correspond to the ten most variable, according to the SSS. **D)** The same miRNA highlighted in panel C were tested in an independent cohort (Iborra M et al., Clin Exp Immunol 2013). The boxplot shows the distribution of the CVs of the Ct values. The most stable miRNAs identified in case study 3 (blue box) have also smallest variation in the validation dataset, and correspondingly the most variable miRNAs (red box) showed highest variability in the validation dataset, supporting the validity of the analysis pipeline.

Figure S3. Case study 4: Healthy donors, different profiling platforms. A) The three scores presented in the text and the SSS are shown in a pairwise scatterplot. Each plot in the lower panel represent a scatterplot of the quantity indicated in the diagonal panel, with points colored according to the platform (Exiqon and TaqMan). In the upper panel the correlation between the score is shown pairwise either overall, or stratifying according to the platform (EX: Exiqon, TM: TaqMan). The rightmost column shows a scatterplot of each score, grouped by platform. The bottommost row show a histogram of the same scores indicated in the diagonal panel, grouped by platform. **B-C)** PCA of miRNA profiled with the TaqMan (B) or Exiqon (C) platorm. The top ten stable miRNA are shown as blue spheres, while the red sphere correspond to the ten most variable, according to the SSS. **D-E)** Cumulative distribution plot of the CVs of the analyzed miRNAs for the TaqMan (D) and Exiqon (E) platform. The presented data are either not normalized (RQ, grey line), normalized with the arithmetic mean (NRQ_mean, yellow line), geometric mean (NRQ_geomean, blue line) or with three stable controls (NRQ_ref, red line). The left-shifted curves shows a reduction on the variability.

Figure S4. Summary of the four cases studies. A radar chart shows the miRNAs that performed better as normalizers in the four case studies. The individual SSS for each miRNAs is shown with a different colored line for the different case studies, as indicated in the legend below the plot. Please note the reverse axis, so that the more stable miRNAs have a score closest to zero. The grey-shaded areas show a summary measure to classify the different reference miRNA. The average of the six SSS is represented with light-grey shade, while the dark-grey shade plot correspond to the "summary of the SSS", i.e. the distance from the origin in 6-dimensional space, using the individual SSS as variables. The latter metric was used to score and summarize the miRNAs. The plot shows that across all case studies, the global normalization measures (median, mean) are the best ranked. Other miRNAs show good performance across different studies, although some variation exists in the corresponding individual rankings

Figure S1

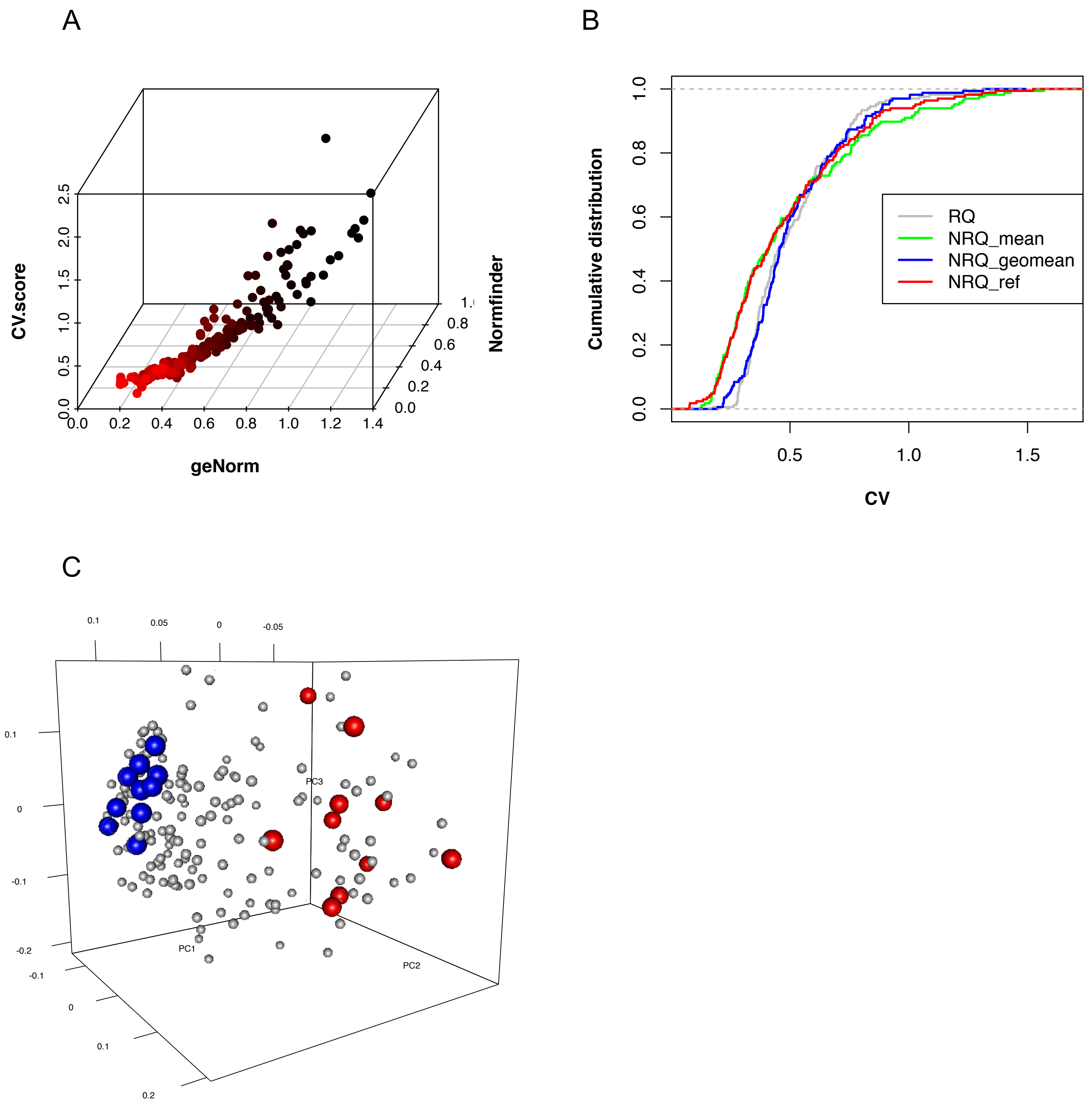
A
CV.score
geNorm
Normfinder
0.0
0.5
1.0
1.5
2.0
2.5
0.2
0.4
0.6
0.8
1.2
1.4
B
Cumulative distribution
CV
RQ
NRQ_mean
NRQ_geomean
NRQ_ref
0.0
0.2
0.4
0.6
0.8
1.0
0.5
1.0
1.5
C
PC1
PC2
PC3
0.1
0.05
0
-0.05
-0.1
-0.2
0.2

Figure S2

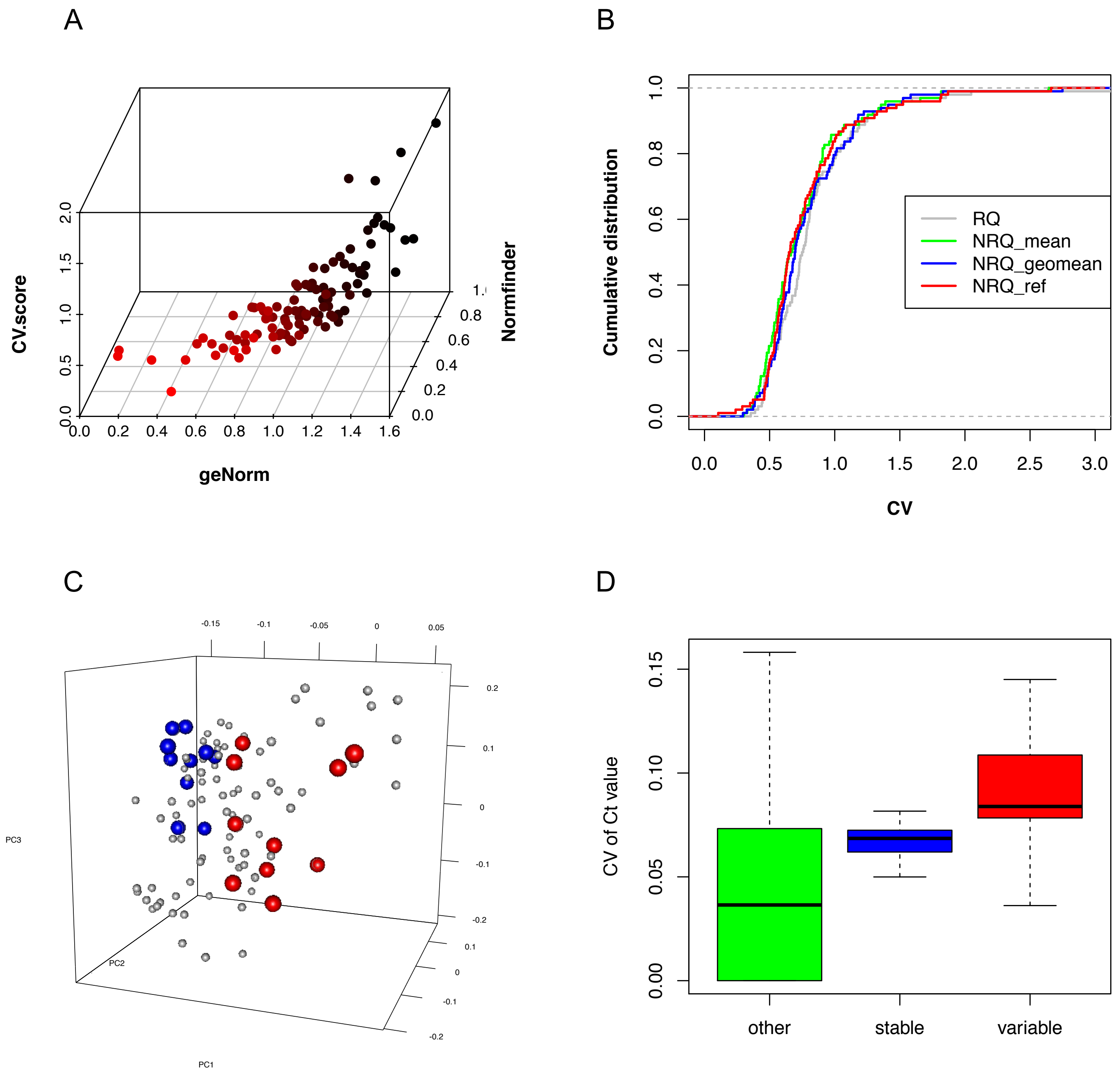
A
CV.score
geNorm
Normfinder
0.0
0.2
0.4
0.6
0.8
1.0
1.2
1.4
1.6
0.5
1.5
2.0
B
Cumulative distribution
CV
RQ
NRQ_mean
NRQ_geomean
NRQ_ref
0.0
0.2
0.4
0.6
0.8
1.0
0.5
1.5
2.0
2.5
3.0
C
PC1
PC2
PC3
-0.15
-0.1
-0.05
0
0.05
0.2
0.1
-0.2
D
CV of Ct value
0.00
0.05
0.10
0.15
other
stable
variable

Figure S3

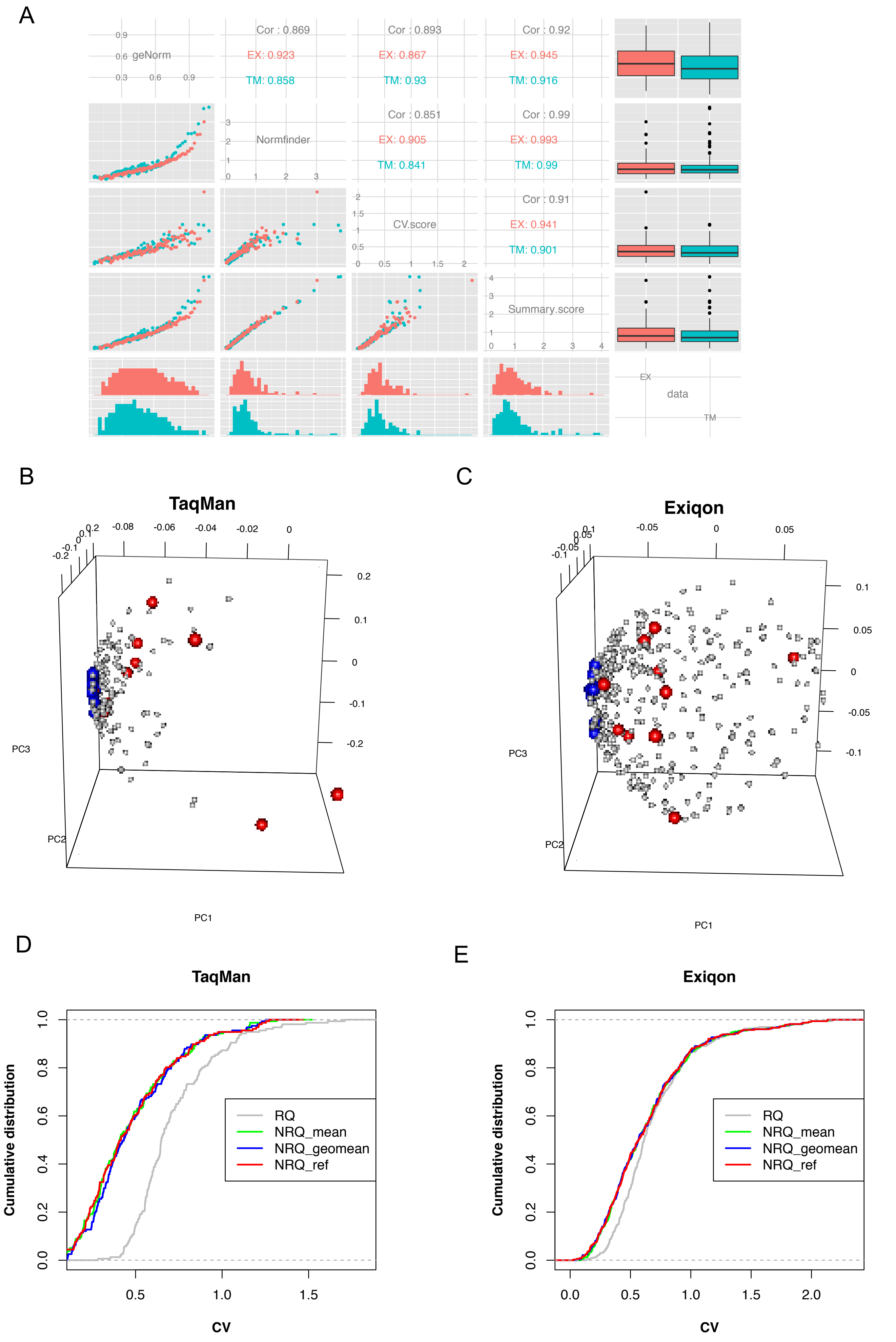
A
Cor : 0.869
EX: 0.923
TM: 0.858
Cor : 0.893
EX: 0.867
TM: 0.93
Cor : 0.92
EX: 0.945
TM: 0.916
geNorm
Cor : 0.851
EX: 0.905
TM: 0.841
Cor : 0.99
EX: 0.993
TM: 0.99
Normfinder
Cor : 0.91
EX: 0.941
TM: 0.901
CV.score
Summary.score
EX
data
TM
B
TaqMan
PC1
PC2
PC3
C
Exiqon
PC1
PC2
PC3
D
TaqMan
Cumulative distribution
CV
RQ
NRQ_mean
NRQ_geomean
NRQ_ref
E
Exiqon
Cumulative distribution
CV
RQ
NRQ_mean
NRQ_geomean
NRQ_ref

Figure S4

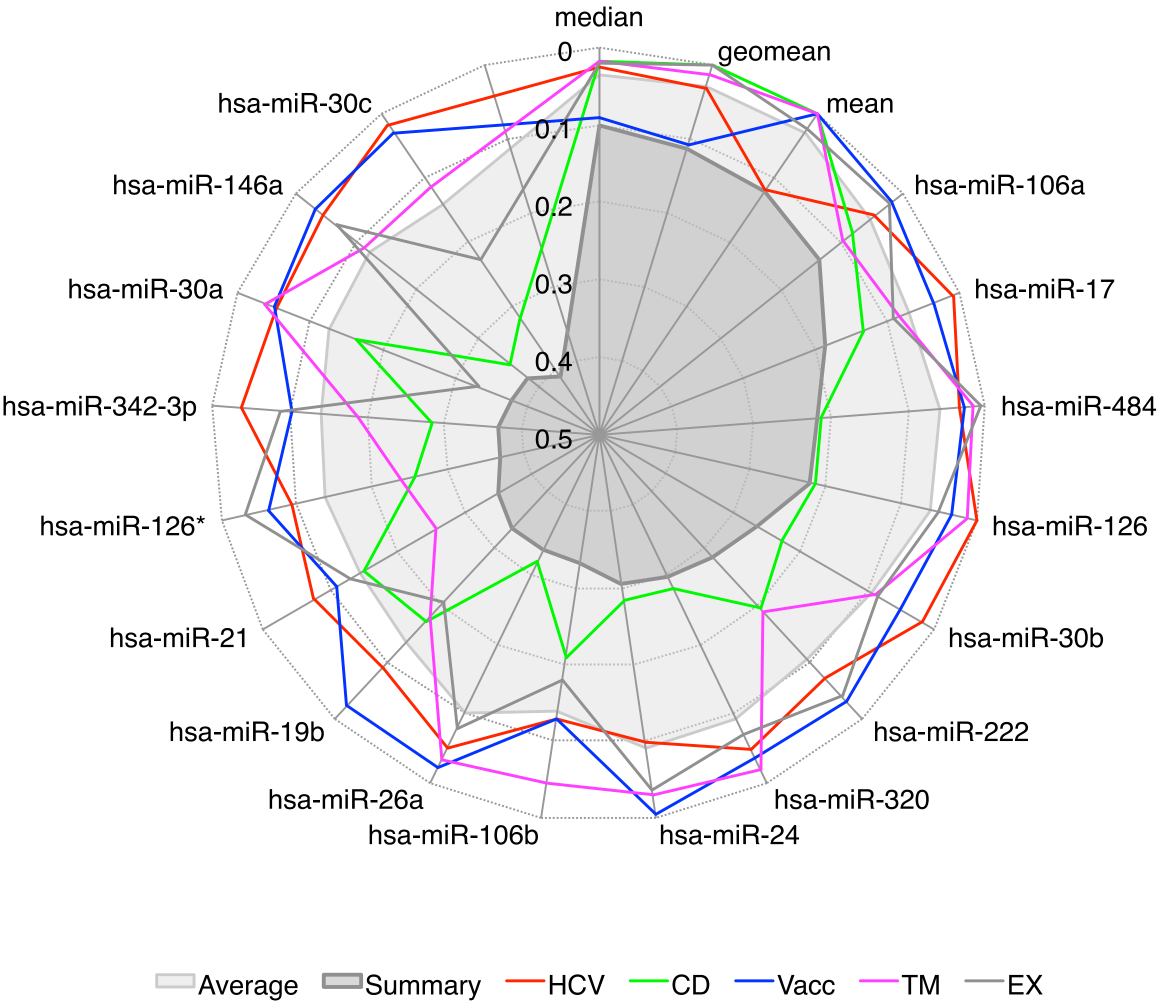
median
geomean
mean
hsa-miR-106a
hsa-miR-17
hsa-miR-484
hsa-miR-126
hsa-miR-30b
hsa-miR-222
hsa-miR-320
hsa-miR-24
hsa-miR-106b
hsa-miR-26a
hsa-miR-19b
hsa-miR-21
hsa-miR-126*
hsa-miR-342-3p
hsa-miR-30a
hsa-miR-146a
hsa-miR-30c
0
0.1
0.2
0.3
0.4
0.5
Average
Summary
HCV
CD
Vacc
TM
EX
